# Stage shift and relative survival for head and neck cancer during the 2020 COVID-19 pandemic: a population-based study of temporal trends

**DOI:** 10.3389/fonc.2023.1253968

**Published:** 2023-09-20

**Authors:** Hanna M. Peacock, Cindy De Gendt, Geert Silversmit, Sandra Nuyts, Jan Casselman, Jean-Pascal Machiels, Francesco Giusti, Bart van Gool, Vincent Vander Poorten, Liesbet Van Eycken

**Affiliations:** ^1^Belgian Cancer Registry, Brussels, Belgium; ^2^Laboratory of Experimental Radiotherapy, Department of Oncology, KU Leuven, Leuven, Belgium; ^3^Department of Radiation Oncology, Leuven Cancer Institute, University Hospitals Leuven, Leuven, Belgium; ^4^Department of Radiology, AZ St-Jan Brugge-Oostende, Bruges, Belgium; ^5^Department of Medical Oncology, Institut Roi Albert II, Cliniques universitaires Saint-Luc, Brussels, Belgium; ^6^Institut de Recherche Clinique et Expérimentale, UCLouvain, Brussels, Belgium; ^7^Otorhinolaryngology-Head and Neck Surgery, University Hospitals Leuven, Leuven, Belgium; ^8^Department of Oncology, Section Head and Neck Oncology, KU Leuven, Leuven, Belgium

**Keywords:** COVID-19, head and neck neoplasm, incidence, mortality, routinely collected health data, delayed diagnosis

## Abstract

**Objective:**

During the first wave of the COVID-19 pandemic in 2020, non-essential health services were suspended in Belgium, and the public was ordered to socially isolate. Underdiagnosis of cancer during this period was reported worldwide. Certain risk factors for head and neck cancer (HNC) overlap with those for COVID-19 incidence and mortality, making underdiagnosis and subsequent stage shift of this potentially rapidly progressing cancer a major concern. We aimed to analyze incidence, clinical stage at presentation, and survival of patients diagnosed with HNC in 2020 in Belgium, considering recent temporal trends.

**Methods:**

Using population-based data from the Belgian Cancer Registry (BCR), we extrapolated 2017-2019 trends in incidence, clinical stage, and 1-year relative survival (1yRS) of HNC to create an expected value for 2020 and compared this to the observed value.

**Results:**

There were 9.5% fewer HNCs diagnosed in 2020, compared to the predicted incidence. Underdiagnosis was larger for males (-11.8%), patients aged 50-64 (-11.2%) and 65-79 (-11.1%), and for oral cavity cancer (-17.6%). Shifts to more advanced stages were observed in larynx and oropharynx tumors and for (male) patients aged 80+. A 2.4 percentage point decline in 1yRS was observed, relative to the increasing trends in 1yRS (2017-2019).

**Conclusion:**

The COVID-19 pandemic led to underdiagnosis of HNC, resulting in shifts to more advanced stage at presentation in certain subgroups. A stage shift can be expected for the 9.5% of tumors not yet diagnosed at the end of 2020. HNC patients diagnosed in 2020 suffered higher than expected mortality.

## 1 Introduction

During the COVID-19 pandemic, access to healthcare services, including cancer diagnosis and treatment, was limited, particularly during the first wave of the pandemic (1 Mar-27 Jun 2020 in Belgium) (1). Otolaryngologists and head and neck surgeons in Europe were advised to take precautions to avoid exposure to COVID-19, including avoiding endoscopic examinations as well as non-urgent and non-cancer surgeries (2). At least one center in Belgium reported severe disruptions to otolaryngology services (3).

Based on accelerated pathology reporting to Belgian Cancer Registry (BCR), we previously estimated that head and neck cancer (HNC) had the largest proportion of missing diagnoses among all tumor types in 2020 (4). Delayed diagnosis of HNC is particularly concerning given that these tumors can double in volume and show nodal progression in as little as one month (5). Single- and multi-center studies from around the world have reported increases in more advanced HNC stage, tumor size, node involvement and/or metastatic disease among these patients as well as increases in emergency presentation during the COVID-19 pandemic (6–12). Population-based analyses have found conflicting results. In the Netherlands, there was no significant shift to more advanced stage at diagnosis for HNC in Jun-Dec 2020 (13). Conversely, in West Scotland, there was a shift towards more advanced stage at diagnosis of HNC in Jun-Oct 2020 (14). To date, little is known about how survival was impacted for HNC patients diagnosed in 2020.

Much research on the impact of the COVID-19 pandemic on cancer compares the pandemic period to the *average* values in previous years, but cancer incidence, stage distribution, and survival are not static. In Belgium, the annual number of diagnoses of HNC for both sexes is increasing (15). Here we examine the incidence, stage distribution and relative survival for HNC in 2020 relative to temporal trends observed in the recent years prior to 2020.

## 2 Materials and methods

### 2.1 Data sources

Data from BCR is estimated to be at least 99% complete for all cancers from 2004 onwards (16); registration is mandatory for all oncological care programs and laboratories for pathological anatomy in Belgium (17). From 2017 onward, UICC TNM8 (18) was used to record tumor stage.

### 2.2 Patient selection

All tumors from the BCR database with incidence 2017-2020, ICD-10 code for HNC (excluding lip; C01-C14;C30-C32) from patients officially residing in Belgium at the time of incidence were included in the study (n=10 582). The following five sub-localizations were considered: oral cavity (C02-C05.0, C06), oropharynx (C01; C05.1-C05.9; C09-C10), hypopharynx (C12-C13), larynx (C32) and other (C07-08;C11;C14;C30-31).

Since surgery with curative intent is performed for fewer than 40% of HNC in Belgium (19), clinical stage (cStage) allows for the broadest comparison of stage at diagnosis. cStage IV tumors were subdivided into those with and without clinically proven distant metastasis (cM1 vs cM0). The few tumor types for which TNM staging was not applicable (n=100; 3.9% of HNC in 2020) were grouped with tumors with unknown cStage.

### 2.3 Statistical methodology

#### 2.3.1 Incidence

To establish the expected incidence for 2020, a linear Poisson count model was calculated over the incidence period 2017-2019 and extrapolated to 2020 using the *proc genmod* procedure (SAS 9.4) (**Supplemental Figure 1A**). This was compared to the observed absolute incidence in 2020 and the difference between the two was taken as significant if the 95% confidence interval (CI) did not contain zero. CIs for the observed values were calculated using the methods of Daly (20), and the CI for the difference was computed by propagating the variance.

Three phases of the pandemic in 2020 were examined separately and compared to the same months in 2017-2019: Jan-Feb (pre-pandemic), Mar-Jun (first wave of the pandemic in Belgium (1)) and Jul-Dec (recovery period: cancer incidence generally returned to baseline from June 2020 (4) despite the start of the second wave of the pandemic in Belgium 31 Aug 2020 (1)).

#### 2.3.2 cStage distribution

To test whether changes in incidence for each cStage group were proportional to the total decline in incidence, a Poisson count model was applied for each cStage separately. The incidence-per-cStage predictions were then multiplied by the overall percentage decline in incidence for all cStages (including unknown stage) to create a corrected-predicted value, per cStage, which was compared to the observed values (**Supplemental Figure 1B**).

There is an annual decline in the percentage of registrations with unknown cStage or where TNM staging is not applicable, from 18.6% in 2017 to 12.0% in 2019 and 9.5% in 2020. Tumors registered with cNx and/or cMx are assumed cN0 and/or cM0, respectively, and a stage is assigned based on the other available cTNM information whenever possible. The assumption that the number of tumors with unknown/not applicable cStage would continue to decline linearly is not considered valid because a certain proportion of tumors will not ever be able to be staged. Therefore, predictions for the combined group unknown/not applicable cStage are not shown; however, these tumors are still included in the overall incidence numbers, used to compute the corrected-predicted value for each individual cStage.

#### 2.3.3 1-year relative survival

One-year survival time was calculated from the date of incidence until the date of death or the last known date alive. One year of follow up was guaranteed for all included patients. 1yRS was calculated as the ratio of the observed survival and the expected survival for a similar group of persons from the general Belgian population (stratified by sex, age, calendar year and geographical region). The Ederer II method (21) was applied to estimate the expected survival using the Belgian national lifetables (22). RS analyses were based on a publicly available algorithm, using time intervals of 1 year wide (23).

A generalized linear model with a Poisson error structure based on collapsed data using exact survival times was used to model the excess hazard (24). A linear association with incidence year over the period 2017-2019 was applied and the projected 1yRS for the year 2020 was used as the expected 1yRS for 2020. The standard errors on the observed and expected 1yRS for the year 2020 were used to compute a z-test to compare the difference in 1yRS estimates.

## 3 Results

### 3.1 Decline in incidence of HNC in 2020

**Figure 1:**
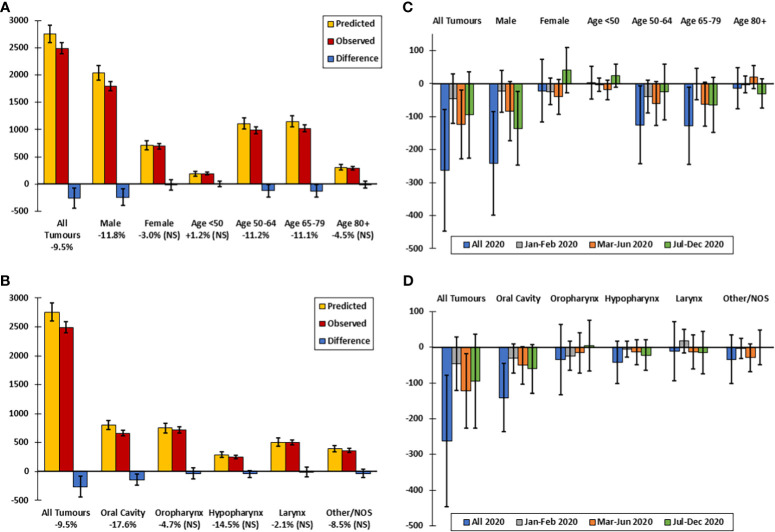
Observed versus predicted incidence of head and neck cancer in Belgium in 2020, by sex, age group and tumor localization. **(A)** Predicted versus observed incidence by sex and age group in 2020. **(B)** Difference between predicted and observed incidence by sex and age group in 2020 and by time period. **(C)** Predicted versus observed incidence by tumor localization. **(D)** Difference between predicted and observed incidence by tumor localization in 2020 and by time period. Predicted values are extrapolated from the incidence trends 2017-2019. Difference between predicted and observed incidence is significant if the 95% confidence interval (error bar) of the difference does not include zero. NS, non-significant.

In 2020 in Belgium, 2491 HNC were diagnosed, which was 9.5% lower than predicted based on the incidence trends 2017-2019 (**Figure 1A**). Patient and tumor characteristics for all years included in the study can be found in **Supplemental Table 1**. The decline was significant in the first wave of the pandemic in Mar-Jun and HNC continued to be underdiagnosed in males in Jul-Dec, with no significant rebounds in diagnosis for any group in this period (**Figure 1B**). The decline in 2020 was much more pronounced in males (n=1799, -11.8%) than in females (n=692, non-significant decline). In the recovery period, Jul-Dec, a significant underdiagnosis remained in males (-13.0%). By age group, the largest declines were seen in the 50-64 (-11.2%) and 65-79 (-11.1%) year age groups. There was a significant underdiagnosis of oral cavity cancer (n=661, -17.6%) but not of other HNC subtypes (oropharynx n= 719, hypopharynx n=249, larynx n=498, other/unspecified n=364; **Figures 1C, D**).

### 3.2 Excess cStage III tumors in males aged 80 and over

**Figure 2:**
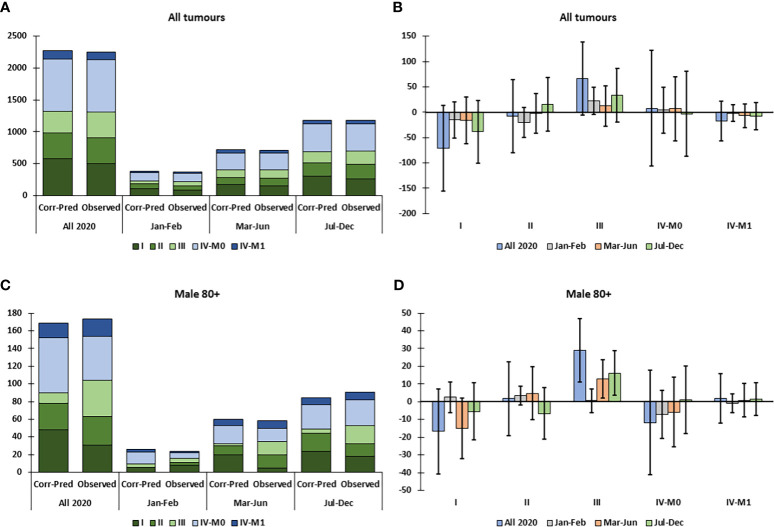
Observed versus predicted incidence by clinical stage of head and neck cancer, corrected for total decline in incidence (Corr-Pred), by sex, age group and tumor localization, in Belgium in 2020. **(A)** Predicted and observed clinical stage distribution for all patients in 2020. **(B)** Difference between observed and corrected-predicted incidence per clinical stage for all patients in 2020 and by time period. **(C)** Predicted and observed clinical stage distribution for males aged 80+ in 2020. **(D)** Difference between observed and corrected-predicted incidence per clinical stage for males aged 80+ in 2020 and by time period. Difference between predicted-corrected and observed incidence is significant if the 95% confidence interval (error bar) of the difference does not include zero.

For all HNC combined, the data give the impression of a decline in cStage I and increase in cStage III tumors particularly in Jul-Dec; however, the incidence by cStage did not differ significantly from the corrected-predicted values (**Figures 2A, B**). Among both sexes and each of the age groups <80 years, the cStage distributions did not differ significantly from the predicted distributions during the COVID-19 pandemic (**Supplemental Figures 2A-E**). In the 80+ age group, there was a significant excess of cStage III tumors in 2020, and particularly after the first wave of the pandemic, Jul-Dec (**Supplemental Figure 2F**) and in males aged 80+ both during (Mar-Jun) and after (Jul-Dec) the first wave (**Figures 2C, D**).

### 3.3 Shifts to increased stage in oropharynx and larynx tumors following the first wave of the pandemic

**Figure 3:**
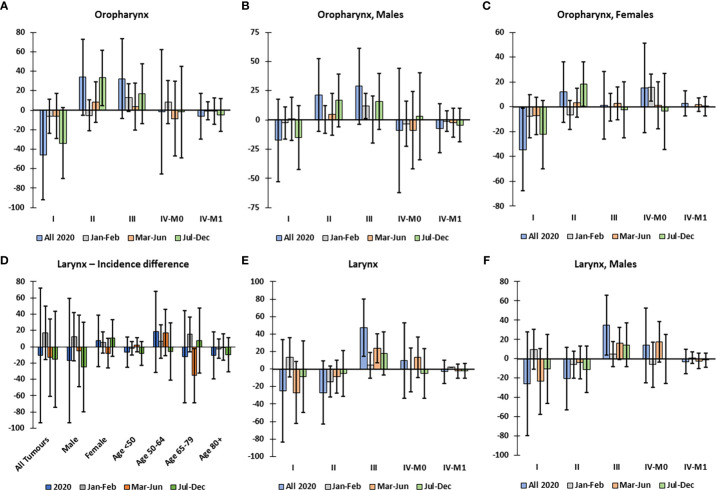
Difference between observed and predicted incidence by clinical stage of oropharynx and larynx tumors, corrected for total decline in incidence, in Belgium in 2020. Difference between observed and corrected-predicted incidence per clinical stage and by time period for **(A)** all oropharynx tumors, **(B)** oropharynx tumors in males, and **(C)** oropharynx tumors in females. **(D)** Difference between observed and predicted incidence of larynx tumors by sex and age group in 2020 and by time period. Difference between observed and corrected-predicted incidence per clinical stage in 2020 and by time period for **(E)** all larynx tumors, and **(F)** larynx tumors in males. Difference between predicted-corrected and observed incidence is significant if the 95% confidence interval (error bar) of the difference does not include zero.

There was no significant decline in the overall incidence of oropharynx or larynx tumors in 2020 (**Figures 1C, D**); however, there were significantly more cStage II oropharynx tumors, specifically in Jul-Dec 2020, while there was an excess decline in cStage I tumors (**Figure 3A**). This trend was not significant in males (**Figure 3B**) but there was a significant excess cStage II in females in Jul-Dec and overall decline in cStage I (**Figure 3C**). The excess incidence of cStage II oropharynx tumors was also observed in the 50-64 age group (not shown). In the 65-79 age group, there was a significant decline in incidence of larynx tumors in Mar-Jun (**Figure 3D**). Overall, in 2020, in Mar-Jun, and specifically in males, there was a significant excess of cStage III larynx tumors (**Figures 3E, F**).

### 3.4 Limited impact on cStage for tumors of oral cavity, hypopharynx and other/unspecified localization

**Figure 4:**
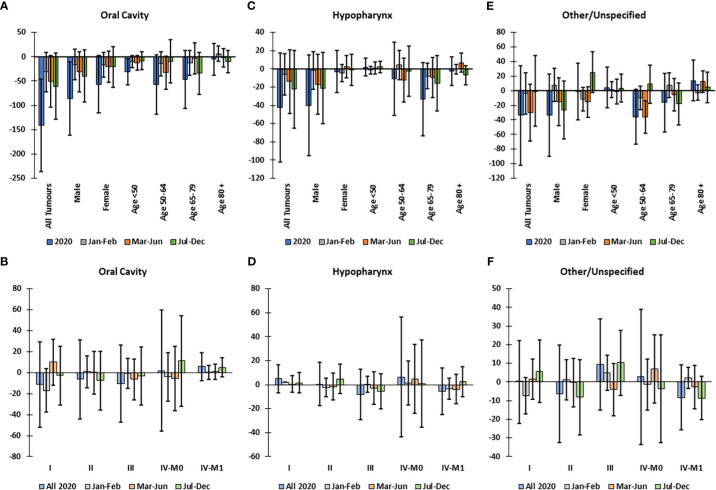
Difference between observed and predicted incidence in 2020 and by clinical stage corrected for total decline in incidence, for oral cavity, hypopharynx and other/unspecified head and neck cancers, in Belgium in 2020. Difference between observed and predicted incidence by sex and age group in 2020 and by time period for **(A)** oral cavity, **(C)** hypopharynx, and **(E)** other/unspecified head and neck tumors. Difference between observed and corrected-predicted incidence per clinical stage in 2020 and by time period for **(B)** oral cavity, **(D)** hypopharynx and **(F)** other/unspecified head and neck tumors Difference between predicted-corrected and observed incidence is significant if the 95% confidence interval (error bar) of the difference does not include zero.

The largest decline in incidence of all subtypes was in oral cavity cancer (**Figure 1C**), which was significant in males (-17.6%) and in patients <50 years old (**Figure 4A**); yet; there was no excess incidence of oral cavity cancer in any cStage overall or when stratified by subgroup (**Figure 4B**). For tumors of the hypopharynx there were no significant declines in incidence (**Figure 4C**) or disproportional changes in cStage (**Figure 4D**). Among tumors with other or unspecified topography, there was a significant decline in incidence in Mar-Jun among patients aged 50-64 (**Figure 4E**); however, the incidence by cStage did not significantly differ from the predicted distribution (**Figure 4F**).

### 3.5 Decline in 1-year relative survival

**Figure 5:**
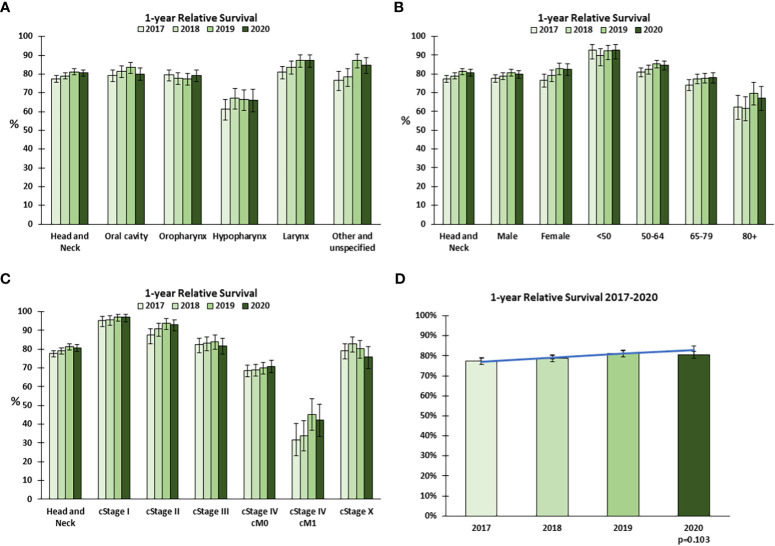
1-year relative survival for head and neck cancer in Belgium in 2020. One-year relative survival for head and neck cancer in 2017-2020 by **(A)** tumor localization, **(B)** sex and age group, and **(C)** clinical stage at diagnosis. **(D)** The trend in 1-year relative survival 2017-2019 was extrapolated to 2020 (predicted, blue line) and compared to the observed (green bars) 1-year Relative Survival. Error bars indicate 95% confidence intervals.

To establish whether the COVID-19 pandemic adversely affected the outcome of patients diagnosed with HNC, we compared the evolution of the 1yRS over the period 2017-2019 with 2020. Over the years 2017-2019 there was an increasing trend in 1yRS, which appeared to stagnate or decline in 2020 (**Figure 5**), particularly for oral cavity tumors (**Figure 5A**), males, patients aged 50-64 years (**Figure 5B**) and cStage III (**Figure 5C**).

To determine whether the 1yRS in 2020 significantly deviated from the trend over the previous years, a linear trend in 1yRS from 2017-2019 was extrapolated to 2020. The 1yRS in 2020 was lower than predicted, though not statistically significantly different (p=0.103, **Figure 5D**); among the 2 393 patients included in the analysis for 2020, there were 62 more excess deaths from HNC within 1 year of diagnosis for patients diagnosed in 2020 relative to the general population (n=459) than would have been predicted based on the trends 2017-2019 (n=397). Given the apparent decline in 1yRS, even within cStages, we examined whether there was a shift in the distribution in cT or cN category, using the same method applied to stage at diagnosis. The data suggest a generally higher cT category overall and a higher cN category in Jul-Dec (**Supplemental Figure 3**).

## 4 Discussion

In Belgium, in 2020, there was a significant decline in the incidence of HNC, particularly oral cavity cancer, and an apparent decline in 1yRS, relative to the trends in previous years. In several subgroups (males [all tumors], oropharynx and larynx tumors), there was a shift to higher cStage, both during and following the first wave of the pandemic.

Underdiagnosis was largest in males and in oral cavity cancer. When controlling for smoking and drinking, males generally have a higher risk of HNC, but sex-disparity is largest in larynx cancer and smallest in oral cavity cancer (25). Furthermore, male patients are more likely to present at advanced stage (26), suggesting a tendency to postpone seeking a diagnosis. Late presentation of HNC is associated with psychological factors such as less optimism, health hardiness and active coping as well as excessive drinking (27), all of which could have been exacerbated by the COVID-19 pandemic. The decline in oral cavity cancer was equivalent in males (-17.6%) and females (-17.9%, not significant) and can largely be attributed to the necessary limitations on dental practices (28). Given that 17.6% of oral cavity cancer and 11.8% of HNC in males remain undiagnosed at the end of 2020, it is unfortunately likely that a shift to more advanced stage will be observed in 2021 data. Based on accelerated pathology reporting to BCR, we expect that HNC also continued to be underdiagnosed during the subsequent waves of the pandemic in 2021 (29).

The magnitude of the decline in diagnosis did not correlate to the stage shift. There was no underdiagnosis of oropharynx and larynx tumors by the end of 2020, yet we observed shifts to more advanced stage in certain subpopulations. For oropharynx tumors, the stage shift did not occur until Jul-Dec 2020, when diagnoses returned to baseline levels, likely indicating progression of tumors after diagnostic delay in the first wave. For larynx tumors, the decline in cStage I and excess in cStage III occurred during the first wave, possibly because tumors with more obvious symptoms were more likely to receive timely diagnosis during the first wave. However, given the rapid growth of HNCs (5), it is also possible that some early stage tumors progressed following a diagnostic delay within the period Mar-Jun.

The observed decline in 1yRS for patients diagnosed in 2020 was not statistically significantly different from the prediction based on trends 2017-2019; however, at a population level the 1yRS for patients diagnosed in 2020 was lower than for 2019 after several years of gains in 1yRS, making this decline in 1yRS a concerning observation. Furthermore, the 1yRS predictions using the trend 2017-2019 may even underestimate the expected 1yRS in 2020, since treatment for patients diagnosed in 2019 also may have been impacted by the COVID-19 pandemic. This observed decline in 1yRS cannot be explained by a shift to more advanced stage, since declines were also apparent in analyses stratified by stage, suggesting that progression of tumor diameter or node involvement within the definition of a specific stage may have taken place. The trend to declining 1yRS specifically in patients with unknown cStage may be related to the improved registration of cStage over the years, such that unknown cStage becomes more restricted to patients with poorer prognosis. Excess mortality may also be associated with treatment delays which could be caused by patients’ fear of contracting COVID-19 by coming into hospital, limited resource availability (e.g., access to surgery), or due to patients contracting COVID-19 and needing to postpone procedures.

### 4.1 Strengths and Limitations

This study was performed using data from a population-based cancer registry covering an entire country, which largely eliminates selection bias that confounds single- and multi-center studies. In 2020, the overlap between registrations of HNC from oncological care programs and laboratories for pathological anatomy (a measure of registration completeness) at BCR was higher than in 2017-2018 and comparable to 2019. We also did not see an increase in non-specific registrations (ICD-10 codes C76.0 or C80) (30).

In this study, we account for temporal trends in cancer incidence and stage distribution. Given the annual increase in cancer incidence (15), taking the average of several previous years as the expected value for 2020 will underestimate the true decline in incidence in 2020. Not only population aging and growth, which could be corrected for by using age-standardized reporting, but also changes in exposure to risk factors and improvements in screening/diagnostic capabilities will impact the incidence and stage at diagnosis of cancer, making it crucial that the complete temporal trends be considered.

COVID-19 infection status may have forced patients to postpone procedures or even have been a direct cause of death. COVID-19 incidence in Belgium was higher in more deprived regions (31) and HNC incidence and mortality is associated with lower socioeconomic position (32, 33). Unfortunately, we lack information on the COVID-19 status of diagnosed or deceased patients (particularly in the first wave of the pandemic, COVID-19 testing was not routinely available). Likewise, patients may have died from COVID-19 before a diagnosis of HNC could be made.

Finally, we are unable to distinguish between HPV-positive and HPV-negative oropharynx tumors in this cohort. HPV-positive oropharyngeal squamous cell carcinomas generally have better overall and progression-free survival than HPV-independent tumors (34). Morphology codes to distinguish HPV-positive and HPV-negative tumors were introduced in 2019 with ICD-O-3.2 (35), and are used in Belgium from 2020. However, it is not possible to compute a predicted value for 2020 since HPV status is not systematically available in the preceding years.

### 4.2 Conclusions

There was substantial underdiagnosis of HNC in Belgium in 2020, considering the recent trends in incidence, particularly for males and oral cavity cancer. Shifts toward higher stage were observed for oropharynx and larynx tumors and for elderly males and there was a reduced 1-year relative survival for HNC patients with diagnosis in 2020. The important impact of the COVID-19 pandemic on HNC diagnosis and outcome should stimulate active planning to ensure continuity of cancer care during future crises affecting the healthcare system. BCR will continue to monitor trends in stage and outcome, as well as treatment patterns, for HNC in the coming years.

## Data availability statement

The cancer cohort data used and analyzed during the study are available from the corresponding author upon reasonable request. The pseudonymized data can be provided within the secured environment of the BCR after having been guaranteed that the applicable GDPR regulations are applied. Requests to access the datasets should be directed to info@kankerregister.org.

## Ethics statement

Ethical approval was not required for the study involving humans in accordance with the local legislation and institutional requirements. Written informed consent to participate in this study was not required from the participants or the participants’ legal guardians/next of kin in accordance with the national legislation and the institutional requirements.

## Author contributions

HP: Conceptualization, Data curation, Formal Analysis, Methodology, Visualization, Writing – original draft, Writing – review & editing. CD: Conceptualization, Methodology, Writing – review & editing. GS: Conceptualization, Formal Analysis, Methodology, Writing – review & editing. SN: Validation, Writing – review & editing. JC: Validation, Writing – review & editing. J-PM: Validation, Writing – review & editing. FG: Conceptualization, Methodology, Writing – review & editing. BG: Conceptualization, Data curation, Methodology, Writing – review & editing. VV: Validation, Writing – review & editing. LE: Conceptualization, Validation, Writing – review & editing.
