# Supplementary material for: Stage shift and relative survival for head and neck cancer during the 2020 COVID-19 pandemic: a population-based study of temporal trends
Source: Front Oncol. 2023 Sep 20;13:1253968. doi: 10.3389/fonc.2023.1253968 (PMC10548264; doi:10.3389/fonc.2023.1253968)
Supplement: Supplementary file 1 [file DataSheet_1.pdf]

Supplementary Material

1 Supplementary Figures and Tables

1.1 Supplementary Figures

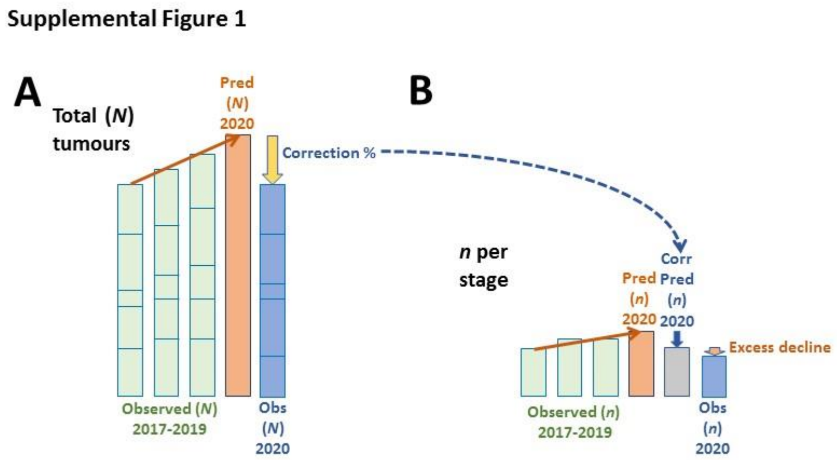

Supplementary Figure 1. Methodology to calculate predicted incidence and stage shift. (A) Poisson count model was used to estimate the average yearly change in cancer diagnoses over the period 2015-2019 and extrapolated to 2020. **(B)** For each subgroup, the Poisson count model was applied independently to establish the predicted value for 2020. The predicted value for each subgroup was "corrected" for the percent decline/increase of the total group. Predicted values were compared to observed values for 2020.

Supplemental Figure 2

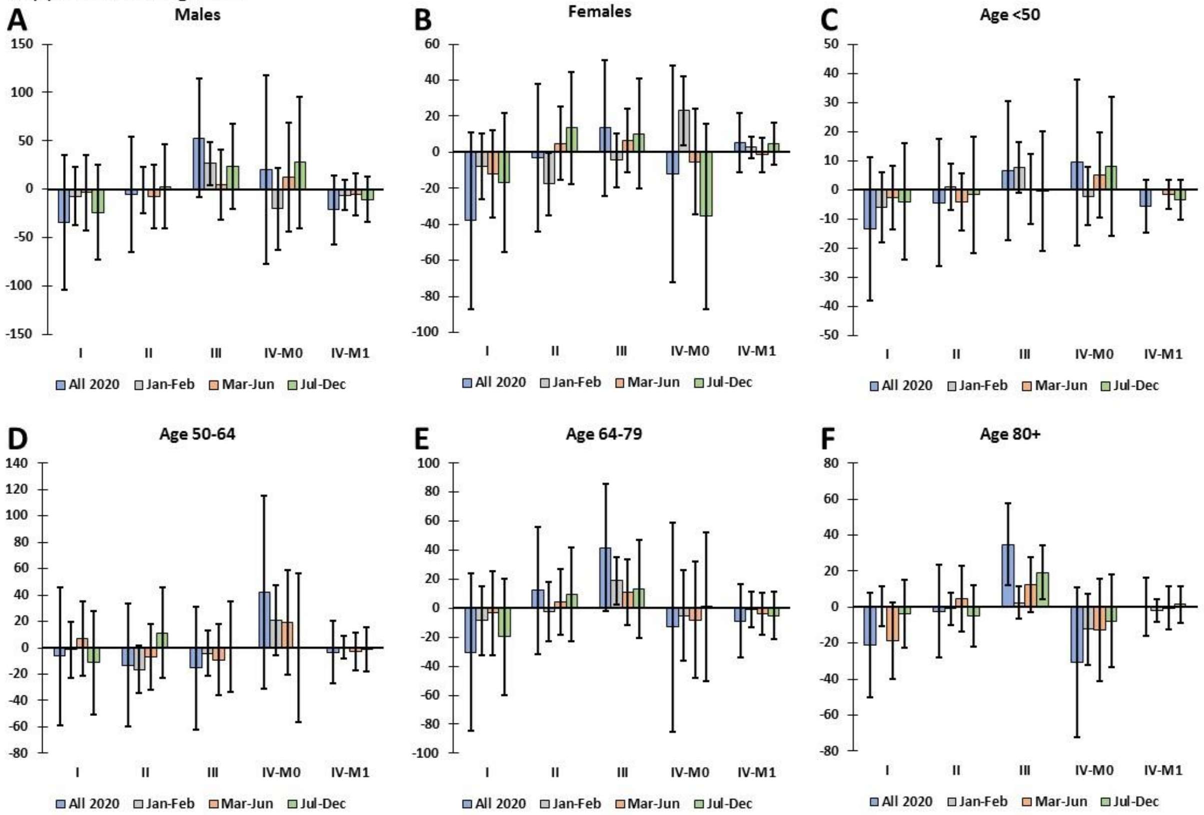

Supplementary Figure 2. Changes in incidence by clinical stage of head and neck cancer in Belgium in 2020, by sex and age group. Difference between observed and corrected-predicted incidence in 2020 and by time period for **(A)** males, **(B)** females, and patients **(C)** aged <50, **(D)** aged 50-64, **(E)** aged 65-79, and **(F)** and aged 80+. Difference between predicted-corrected and observed incidence is significant if the 95% confidence interval (error bar) of the difference does not include zero.

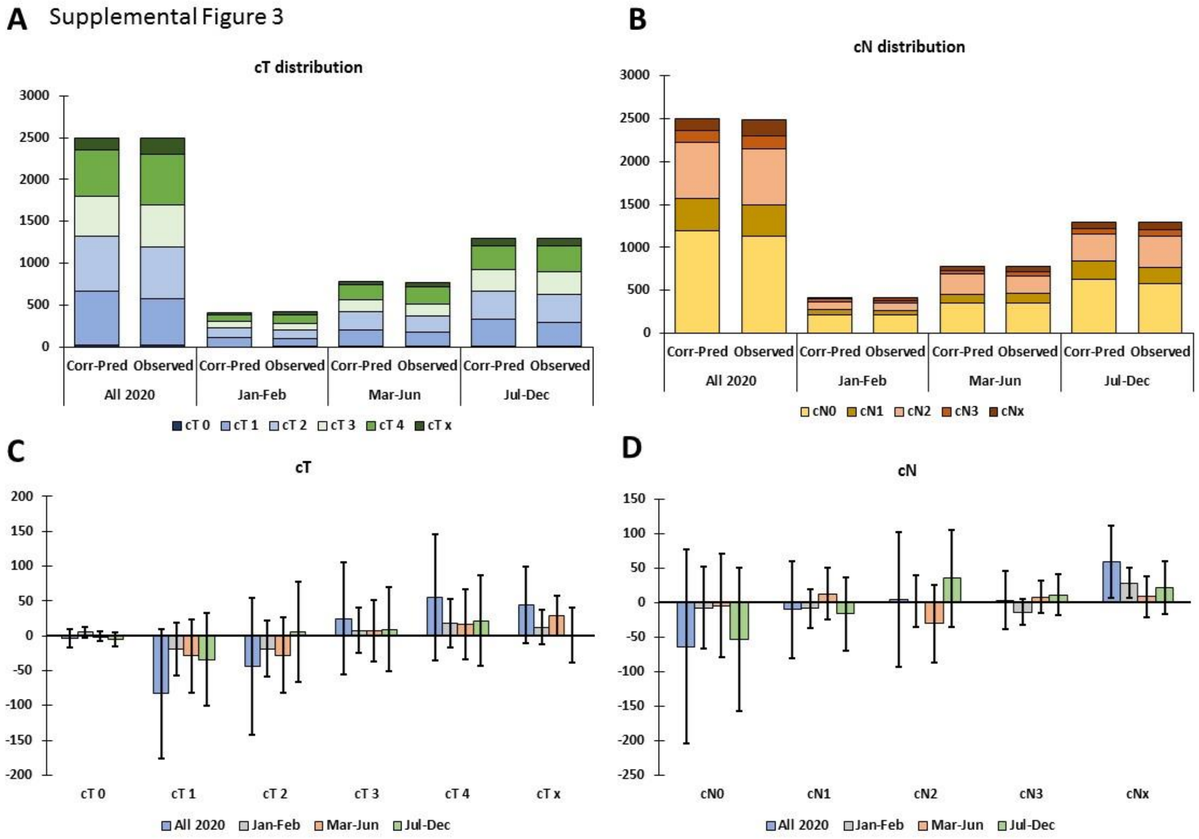

Supplementary Figure 3. Observed versus corrected-predicted tumor diameter and node involvement. Clinical **(A)** T category and **(B)** N category distribution predictions and observed distributions corrected for incidence decline (Corr-Pred) for head and neck cancer in 2020 and by time period. Difference between observed and corrected-predicted incidence per clinical **(C)** T category and (D) N category for head and neck cancerin 2020 and by time period. Difference between predicted-corrected and observed incidence is significant if the 95% confidence interval (error bar) of the difference does not include zero.

1.2 Supplementary Tables

Supplementary Table 1. Patient and tumor characteristics.

	Overall		2017		2018		2019		2020	
	N	%	N	%	N	%	N	%	N	%
**Total**	**10 582**		**2 671**		**2 692**		**2 728**		**2 491**	
**Sex**										
Male	7 759	*73.3*	1 977	*74.0*	1 952	*72.5*	2 031	*74.5*	1 799	*72.2*
Female	2 823	*26.7*	694	*26.0*	740	*27.5*	697	*25.5*	692	*27.8*
**Mean Age (SD)**	**65**	***(11.9)***	**64**	***(11.7)***	**64**	***(11.6)***	**65**	***(11.7)***	**65**	***(11.5)***
**Age group**										
<50 years	784	*7.4*	203	*7.6*	196	*7.3*	194	*7.1*	191	*7.7*
50-64 years	4 445	*42.0*	1 179	*44.1*	1 141	*42.4*	1 138	*41.7*	987	*39.6*
64-79 years	4 161	*39.3*	988	*37.0*	1 058	*39.3*	1 092	*40.0*	1 023	*41.1*
80+ years	1 192	*11.3*	301	*11.3*	297	*11.0*	304	*11.1*	290	*11.6*
**Topography**										
Oral cavity	2 796	*26.4*	670	*25.1*	704	*26.2*	761	*27.9*	661	*26.5*
Oropharynx	3 057	*28.9*	787	*29.5*	789	*29.3*	762	*27.9*	719	*28.9*
Hypopharynx	1 135	*10.7*	300	*11.2*	290	*10.8*	296	*10.9*	249	*10.0*
Larynx	2 197	*20.8*	599	*22.4*	559	*20.8*	541	*19.8*	498	*20.0*
Other and Unknown	1 397	*13.2*	315	*11.8*	350	*13.0*	368	*13.5*	364	*14.6*
**Clinical Stage***										
I	1 904	*20.8*	395	*18.2*	439	*19.0*	567	*23.6*	503	*22.3*
II	1 514	*16.6*	332	*15.3*	379	*16.4*	406	*16.9*	397	*17.6*
III	1 645	*18.0*	418	*19.2*	430	*18.6*	387	*16.1*	410	*18.2*
IV-M0	3 586	*39.2*	925	*42.5*	930	*40.3*	907	*37.8*	824	*36.5*
IV-M1	490	*5.4*	105	*4.8*	130	*5.6*	134	*5.6*	121	*5.4*
X/NA	1 443	*13.6*	496	*18.6*	384	*14.3*	327	*12.0*	236	*9.5*

**Percentages for known clinical stage are computed excluding X/NA*
